# Exceptionally High-Temperature-Resistant Kapton-Type Polyimides with *T*_g_ > 520 °C: Synthesis via Incorporation of Spirobis(indene)-bis(benzoxazole)-Containing Diamines

**DOI:** 10.3390/polym17070832

**Published:** 2025-03-21

**Authors:** Peng Xiao, Xiaojie He, Qinghua Lu

**Affiliations:** 1 Institute of Micro/Nano Materials and Devices, Ningbo University of Technology, Ningbo 315211, China; pengxiao2020@tongji.edu.cn; 2 School of Chemical Science and Technology, Tongji University, Siping Road No. 1239, Shanghai 200092, China; 3 State Key Laboratory of Synergistic Chem-Bio Synthesis, School of Chemistry and Chemical Engineering, Shanghai Jiao Tong University, Dongchuan Road No. 800, Shanghai 200240, China

**Keywords:** Kapton-type polyimides, spirobis(indene)-bis(benzoxazole), ultra-high *T*_g_, good mechanical properties, molecular dynamics simulations

## Abstract

Polyimides (PIs), recognized for their exceptional thermal stability, are extensively employed in advanced applications, including aerospace, flexible displays, flexible solar cells, flame-retardant materials, and high-temperature filtration materials. However, with the continuous advancements in science and technology, the demand for improved thermal performance of PIs in these application areas has increased significantly. In this study, four spirobis(indene)-bis(benzoxazole) diamine monomers (5a, 5aa, 5b and 5bb) were designed and synthesized. These monomers were copolymerized with pyromellitic dianhydride (PMDA) and 4,4-diaminodiphenylmethane (ODA) to develop Kapton-type PIs. By varying the copolymerization molar ratios of the different diamines, a series of novel ultrahigh-temperature-resistant PI films were successfully prepared, and it was found that incorporating a highly rigid and twisted spirobis(indene)-bis(benzoxazole) structure into the PI matrix enhances the rigidity of the polymer chains and restricts their mobility, thereby significantly improving the thermal performance of the PI films. When 5a and ODA were copolymerized at molar ratios of 1:9 and 4:6, the glass transition temperature (*T*_g_) of Kapton-type films significantly increased from 396 °C to 467 °C and >520 °C, respectively. These PI films also exhibit exceptional mechanical properties, with the modulus increasing from 1.6 GPa to 4.7 GPa, while demonstrating low dielectric performance, as evidenced by a decrease in the dielectric constant (*D*_k_) from 3.51 to 3.08 under a 10 GHz high-frequency electric field. Additionally, molecular dynamics simulations were employed to further explore the relationships between polymer molecular structure, condensed states, and film properties, providing theoretical guidance for the development of polymers with ultrahigh thermal resistance and superior overall performance.

## 1. Introduction

With the increasing demand for heat resistance in fields such as aerospace [1,2], flexible displays, flexible solar cells [3,4], fireproof materials, and high-temperature filtration materials [5], there has been a growing focus on the research and development of high-temperature-resistant materials. Compared to metallic materials and inorganic non-metallic materials, polymer materials offer significant advantages such as high specific strength, excellent insulation, and high elasticity [6,7,8,9,10]. Among the various high-temperature-resistant polymer materials, polyphenylene sulfide (PPS) [11], polyaryletherketone (PAEK) [12,13], polysulfone (PSU) [14,15], polyarylate (PAR) [16], polybenzoxazole (PBO) [17,18], polyamide (PA) [19,20], and liquid crystal polymers (LCP) [21,22] have been extensively studied and widely applied. However, these polymeric materials generally struggle to withstand temperatures exceeding 400 °C, thereby limiting their potential applications under extreme thermal conditions. PI is considered one of the most heat-resistant polymer materials due to its excellent combination of properties, including resistance to high and low temperatures, high strength, high modulus, and radiation resistance [23,24,25], along with strong design flexibility and processing diversity [26,27], making it increasingly prominent in both scientific and engineering applications.

The homobenzene-based Kapton^®^ series of films, initially developed by DuPont, have since been improved to include new models such as Kapton^®^ PV, Kapton^®^ FCR, Kapton^®^ HN, and Kapton^®^ MT+. Due to their low production cost, mature processing technology, and outstanding comprehensive performance, Kapton^®^ films have become the most widely commercially applied high-temperature polymers to date [28,29,30]. In recent years, the rapid development in related fields has driven the urgent need for ultra-high-temperature-resistant PI materials. For instance, flexible CIGS solar cell substrates require deposition temperatures exceeding 500 °C to produce high-quality absorber layers [3,4,31]. Currently, almost all PI films have difficulty tolerating temperatures above 500 °C, severely limiting the conversion efficiency of solar cells. Consequently, developing PI films with ultra-high thermal resistance (*T*_g_ > 500 °C) while maintaining good thermal dimensional stability and mechanical properties is of significant research interest.

Typically, the *T*_g_ of polymers is closely related to their molecular structure and interchain interactions. Factors influencing molecular structure include rigidity, the rotational freedom of chains, and steric hindrance effects. Generally, the greater molecular rigidity and reduced rotational freedom of bonds can effectively hinder the movement of PI molecular chains, thereby increasing the *T*_g_ of PI films. For example, introducing nitrogen-containing aromatic heterocyclic structures such as benzoxazole [32], quinoxaline [33], pyrimidine [34], and carbazole [35] into the PI structure can effectively enhance the rigidity and linear alignment of the polymer chains, improving the thermal resistance and dimensional stability of PI. Additionally, incorporating rigid, twisted structures such as spiro [36,37], fluorene [38,39], adamantane [40], and carborane [41] into the PI structure can effectively limit molecular chain mobility and increase steric hindrance, enhancing both the thermal resistance and transparency of polymer films. Moreover, introducing hydrogen bonds or crosslinking structures between molecular chains can enhance interchain interactions, further improving PI’s thermal resistance [23,42,43], although often at the expense of mechanical properties.

In this study, we simultaneously introduced a rigid rod-like benzoxazole structure and a rigid twisted spirobis(indene) structure into the diamine monomer, designing and synthesizing four isomeric diamine monomers containing fused benzoxazole groups. Through copolymerization, these spiro-fused benzoxazole diamines were incorporated into Kapton-type PIs in varying molar ratios. The introduction of the benzoxazole and spiro structures significantly enhanced the rigidity and torsional properties of the resulting polymer chains, yielding PI films with excellent thermal resistance. When the molar ratio of 5a/ODA was just 1:10, the *T*_g_ of the resulting PI film was raised from 396 °C (Kapton) to 467 °C, and the initial modulus increased from 1.6 GPa to 2.5 GPa. At molar ratios of 2:8 and 4:6, the corresponding PI films exhibited *T*_g_ values of 500 °C and 520 °C, with the initial modulus reaching 3.3 GPa and 4.7 GPa, respectively. The other three isomers (5aa, 5b, 5bb) also demonstrated similarly excellent thermomechanical properties. Furthermore, the introduction of the spiro structure effectively reduced the molar polarizability of the polymers, resulting in a low dielectric constant (3.08 at 10 GHz). Additionally, molecular dynamics simulations were used to further investigate the relationship between the molecular structure of PI and its film properties, ultimately providing a viable strategy for the design and preparation of ultra-heat-resistant and high-modulus PI films.

## 2. Experimental Section

### 2.1. Materials and Characterization

In this paper, all the materials employed, detailed characterization methods, and specific processes of the related simulations [44] are comprehensively described in the Supplementary Materials.

### 2.2. Monomer Synthesis

The synthesis route for isomeric diamine monomers containing spirobis(indene)-bis(benzoxazole) is depicted in Scheme S1. Starting with bisphenol A, the synthesis involves four reaction steps: (1) isomerization; (2) nitration; (3) reduction; and (4) dehydration cyclization. This process yields four isomeric diamines (5a, 5aa, 5b and 5bb) with good yields. Detailed synthesis procedures can be found in our previous reports [36].

### 2.3. Preparation of Polyimide Films

**Scheme 1:**
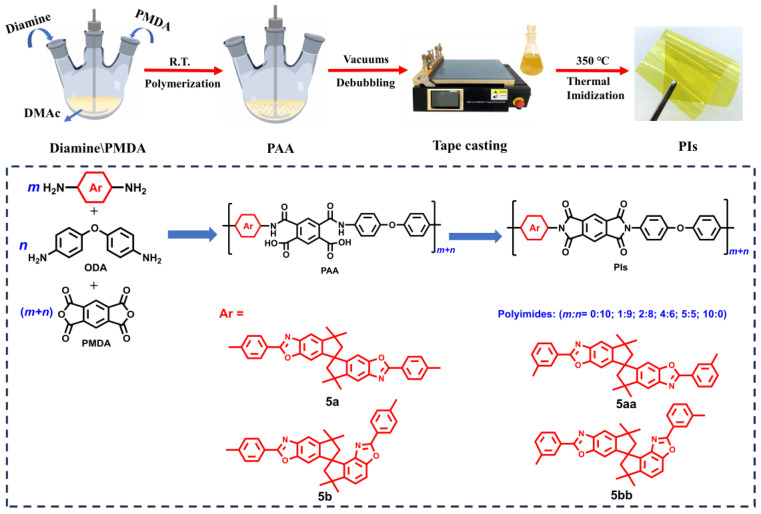
Synthesis of Kapton-type PIs.

All PIs were synthesized using the two-step thermal imidization method, as illustrated in Scheme 1. For instance, in the case of 5a4-ODA6-PMDA, the two diamines 5a and ODA are combined in a molar ratio of 4:6, followed by copolymerization with the anhydride PMDA at a 1:1 ratio. The specific experimental steps are as follows: a 100 mL three-neck flask and a Teflon stirrer were placed in a vacuum oven at 80 °C for approximately 15 min. The flask and stirrer were then transferred to a mechanical stirring apparatus and connected to a nitrogen gas line. Under nitrogen atmosphere at room temperature, diamine 5a (1.7990 g, 3.33 mmol) and ODA (1.0000 g, 5.00 mmol) were added, followed by 5 mL of DMAc solvent. The mixture was stirred until the diamines completely dissolved, after which anhydride PMDA (1.8169 g, 8.33 mmol) was added, along with an additional 10 mL of DMAc solvent. The stirring speed was set to 100–150 r/min. The viscosity of the solution gradually increased, and stirring continued for 12 h. The resulting yellow transparent poly(amic acid) (PAA) solution was then placed in a vacuum oven at room temperature for over 10 h to degas, preparing it for the next step.

PI films were prepared using the casting method. In a cleanroom with humidity maintained below 35%, clean and dry glass plates (20 × 25 cm) were placed on an automatic film coater. The degassed PAA solution was poured onto the glass plates, and the blade height was adjusted to 600 μm, allowing the coating to proceed at a speed of 5 mm/min to ensure an even application of the solution. The glass plates were then placed in a vacuum oven to remove the solvent, first at 80 °C and then at 120 °C, for 2 h each. Following this, the plates were transferred to a high-temperature oven under an air atmosphere, where they were held at 150 °C, 200 °C, 250 °C, and 300 °C for 1 h each, culminating in a final hold at 350 °C for 1 h, with a heating rate of 5 °C/min. Once the oven cooled to below 50 °C, the glass plates containing the PI films were removed and immersed in hot water for peeling. The films were then rinsed with ultrapure water and dried in a vacuum oven at 80 °C for 1 h. The final product was a gold-colored PI film with a thickness of 30–45 μm.

## 3. Results and Discussion

### 3.1. Characterization of Polyimides

This study selected the most common commercial Kapton system (ODA-PMDA) to copolymerize with four isomeric monomers (5a, 5aa, 5b and 5bb), resulting in a series of PI films. The molecular weights and distributions of the intermediates, poly(amic acid) (PAA), were determined using solution permeation chromatography (GPC), with specific values provided in Table 1. The film-forming characteristics of these PIs were also documented. All PAAs exhibited high molecular weights, indicating the high reactivity and purity of the four isomeric diamines. When the molar ratio of the four diamines to ODA exceeds 1:1 and copolymerizes with PMDA, the synthesized PAA, despite achieving a sufficiently high molecular weight, fails to form self-supporting films after high-temperature imidization due to the excessive rigidity of the polymer. However, when the ratio of the synthesized monomers to ODA was maintained at no more than 4:6, the resulting PI films demonstrated good flexibility, as shown in the optical photographs of the films in Figure S1.

**Figure 1:**
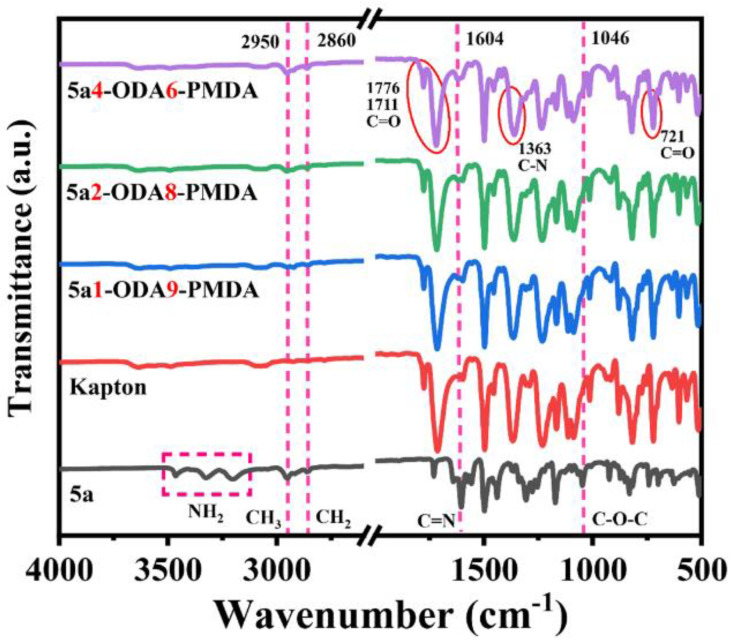
FTIR spectra of 5a and Kapton-type PIs.

The yellow PI films obtained were characterized by Fourier-transform infrared spectroscopy (FT-IR), as shown in Figure 1. The infrared spectra of copolymerized PI films with varying amounts of monomer 5a (0:10, 1:9, 2:8 and 4:6) are presented. The peaks at 1776 cm^−1^ and 1711 cm^−1^ correspond to the asymmetric and symmetric stretching vibrations of the C=O bonds in the imide rings, respectively. The peak at 1363 cm^−1^ is attributed to the C–N stretching vibration in the imide rings, while the peak at 721 cm^−1^ represents the bending vibration of C=O in the imide rings. Peaks at 2950 cm^−1^ and 2711 cm^−1^ are associated with the C–H stretching vibrations of CH_3_ and CH_2_ groups on the spirobis(indene) structure. Additionally, the peak at 1604 cm^−1^ corresponds to the C=N stretching vibration in the benzoxazole ring, and the peak at 1046 cm^−1^ corresponds to the C–O–C stretching vibration in the benzoxazole ring. Notably, no absorption peak was observed around 1650 cm^−1^ (indicative of the C=O stretching vibration in the intermediate poly(amic acid)), confirming that the spirobis(indene)-bis(benzoxazole) structures were successfully integrated into the PI molecular chains, and indicating that the PI films were fully imidized.

### 3.2. XRD Analysis

**Figure 2:**
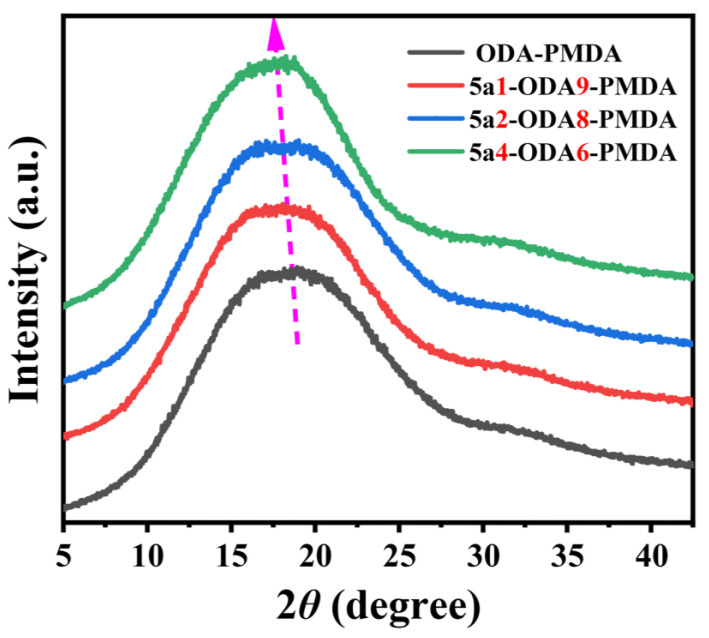
XRD profiles of Kapton-type PIs.

The packing state of polymer molecular chains significantly influences the physicochemical properties of polymers. Here, four types of Kapton-type PI films with varying 5a contents (ranging from ODA-PMDA to 5a4-ODA6-PMDA) were characterized using XRD, as shown in Figure 2. All polymers displayed a broad peak at approximately 2θ ≈ 18°, indicating that the PIs are amorphous. Based on Bragg’s equation, the calculated inter-chain distances for ODA-PMDA to 5a4-ODA6-PMDA were 4.72 Å, 4.87 Å, 4.94 Å, and 5.10 Å, respectively. The increasing inter-chain distances with higher 5a monomer content in Kapton are primarily attributed to the high rigidity and twisted structure of the spirobis(indene)-benzoxazole units. Additionally, the densities of these four PI films were measured, yielding values of 1.402 g/cm^3^, 1.391 g/cm^3^, 1.375 g/cm^3^, and 1.358 g/cm^3^ for ODA-PMDA to 5a4-ODA6-PMDA, respectively. The combined analysis of XRD and density results consistently indicated that the introduction of the 5a monomer leads to an increase in the free volume of Kapton-type PIs.

### 3.3. Thermal Properties

**Figure 3:**
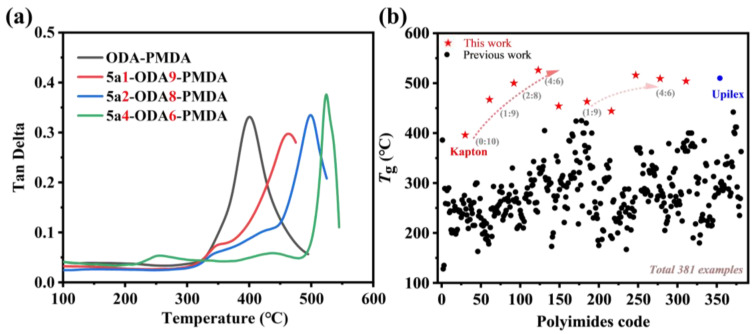
(**a**) DMA curves of Kapton-type PIs; (**b**) our work compares the *T*_g_ values of PI films with those previously reported [45].

The *T*_g_ of a polymer, which is a key characteristic parameter, governs its processing and performance properties. The *T*_g_ values of all PI films were characterized by dynamic mechanical analysis (DMA) (as shown in Figure 3a and Figure S2), with detailed data provided in Table 2. All films demonstrated remarkably high *T*_g_ values. The *T*_g_ of the Kapton film (ODA-PMDA) was measured to be 396 °C. When the molar ratios of 5a to ODA were adjusted to 1:9, 2:8 and 4:6, the *T*_g_ values of the resulting PI films increased to 467 °C, 500 °C, and 520 °C, respectively, exceeding nearly all previously reported *T*_g_ values for PI films in the literature (Figure 3b). The introduction of just 10% or 20% of the 5a monomer resulted in *T*_g_ increases of 71 °C and 104 °C for the Kapton films, respectively, demonstrating that incorporating the highly twisted, rigid spirobis(indene)-bis(benzoxazole) structures into the PI backbone effectively restricts the mobility of PI molecular chains, leading to a substantial enhancement in *T*_g_. Similarly, copolymerizing the other three isomeric diamines (5aa, 5b, 5bb) at a 1:9 molar ratio yielded *T*_g_ values of 454 °C, 463 °C and 444 °C, respectively. When copolymerized at a 4:6 ratio, the *T*_g_ values of the corresponding films were 510 °C, 509 °C and 504 °C, respectively, indicating that these other three isomeric diamines also significantly improve the *T*_g_ of the PI films. Among the four polymer isomers, the polymer with symmetric isomers of the amino group and oxazole ring (5a series PI) exhibited the highest *T*_g_. Conversely, the polymer with meta-isomers of the amino group and asymmetric isomers of the oxazole ring (5bb series PI) showed the lowest *T*_g_. The *T*_g_ values of the corresponding isomeric PIs followed the order 5a > 5aa ~ 5b > 5bb.

**Figure 4:**
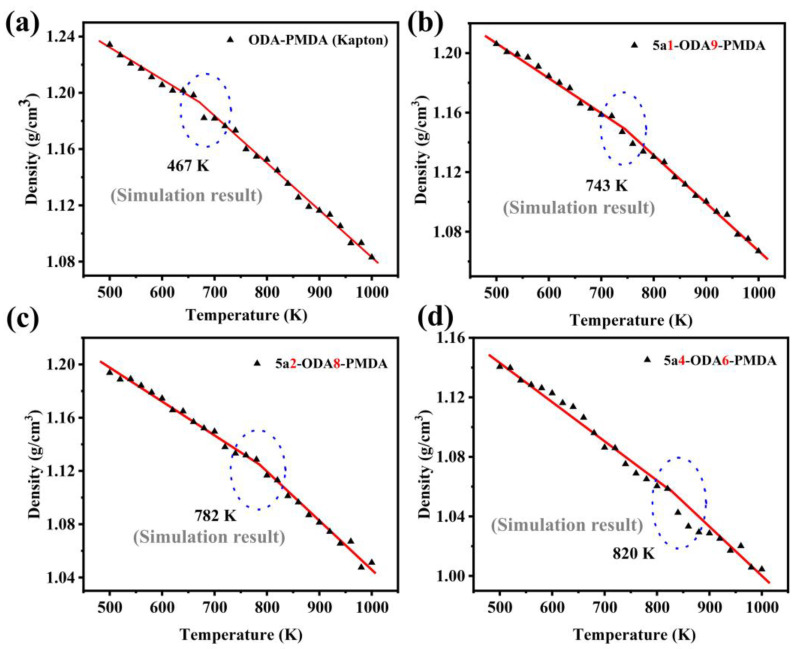
(**a**–**d**) Plot of density vs. temperature.

When a polymer is below its *T*_g_, the motion of the polymer molecular chains is restricted, and the segments remain in a frozen state, rendering the polymer in a glassy state. As the temperature increases and reaches the *T*_g_, the motion of the polymer segments is activated, and the internal segments begin to move. At this point, the polymer is in a rubbery state, with noticeable changes in both its free volume and density. This allows for the simulation of the polymer’s aggregation state at different temperatures. Subsequently, the density parameters can be calculated, and a relationship graph between the polymer temperature and density can be plotted, from which the theoretical *T*_g_ can be determined at the point of the abrupt change. In the temperature–density curve of the polymer, the point of the most pronounced property change corresponds to the theoretically calculated *T*_g_ [44,46,47,48]. Here, molecular dynamics simulations of PI were performed using Materials Studio 2019 with a temperature range of 1000 K to 500 K, decreasing by 20 K per step. At each temperature point, the system was first stabilized under NVT (number of particles, volume, temperature) conditions, followed by equilibration under NPT (number of particles, pressure, temperature) conditions to obtain density data. The mean and standard deviation of the density were computed, and the COMPASSII force field was employed to accurately capture the temperature-dependent density characteristics. Detailed modeling and computational procedures can be found in the Supporting Information. Subsequently, a correlation plot between the temperature and density of PI was generated. Linear regression fitting was conducted for data points from both the low-temperature and high-temperature regions. The results, as shown in Figure 4, indicate that the theoretical *T*_g_ values for these PI films are 675 K (402 °C), 743 K (470 °C), 782 K (509 °C), and 820 K (547 °C), respectively. These values demonstrate good consistency with the experimental trends observed for these PI samples, as presented in Table 2.

**Figure 5:**
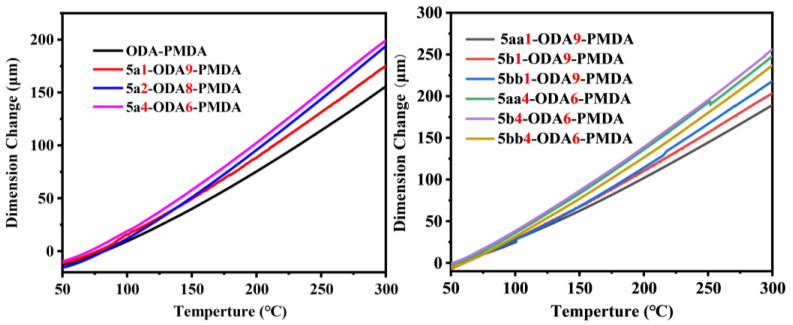
TMA curves of Kapton-type PIs.

The thermal dimensional stability of the PI films containing spirobis(indene)-bis(benzoxazole) was characterized using thermomechanical analysis (TMA). Figure 5 presents the TMA test curves for all PI films. The coefficient of thermal expansion (CTE) for all films are listed in Table 2. As the molar ratio of 5a in the copolymerization increased from 0 to 1:9, 2:8 and 4:6, the CTE of the films exhibited a slight increase, rising from 32 to 34, 36 and 38 ppm/K, respectively. For the other three isomeric diamines (5aa, 5b, 5bb) copolymerized at a 1:9 molar ratio, the corresponding CTE values were 34, 36 and 39 ppm/K, respectively. Additionally, when copolymerized with PMDA at a 4:6 ratio, the CTE values of the resulting films were 43, 45 and 42 ppm/K, respectively. The magnitude of the CTE is determined by the intrachain packing of the polymer chains, which is influenced by factors such as molecular rigidity, linearity, interchain interactions, and processing conditions. The spirobis(indene) structure provides a highly twisted rigid framework, while the benzoxazole contributes a rigid, rod-like linear structure. The experimental results indicate that the simultaneous incorporation of both structures into ODA-PMDA type PI only slightly increases the CTE value, primarily due to the rigidly twisted structure of spirobis(indene). Fortunately, this does not significantly compromise the thermal dimensional stability of the polymer films. A comparison of the four isomeric PIs reveals that the para-isomer of the amino group combined with the symmetric isomer of the oxazole ring (5a series PI) exhibits superior thermal dimensional stability, likely attributed to the higher linearity of its molecular chains [36,49].

**Figure 6:**
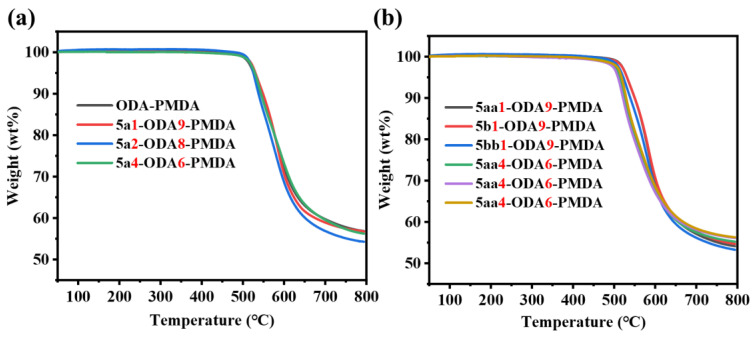
(**a**,**b**) TGA curves of Kapton-type PIs.

The thermal stability of the films was characterized using thermogravimetric analysis (TGA), with the TGA decomposition curves presented in Figure 6 and corresponding values listed in Table 2. As the copolymerization ratios of 5a and ODA increased from 0:1 to 1:9, 2:8 and 4:6, the thermal stabilities of the films showed slight reductions, but were still sufficiently thermally stable. Specifically, the temperature for 5% weight loss (*T*_d_^5%^) decreased from 558 °C to 537 °C, 525 °C and 522 °C, while the temperature for 10% weight loss (*T*_d_^10%^) decreased from 581 °C to 549 °C, 539 °C and 535 °C. This reduction in *T*_d_^5%^ and *T*_d_^10%^ is attributed to the susceptibility of the four methyl groups in the spirobis(indene) structure to high-temperature decomposition. The residual weight at 800 °C (*R*_w_) for the films remained stable, ranging from 56.8% to 56.2%, indicating that the incorporation of spirobis(indene)-bis(benzoxazole) structures does not adversely affect the *R*_w_. For the other three isomeric diamines (5aa, 5b, 5bb) copolymerized at a 1:9 molar ratio, the corresponding PI films exhibited *T*_d_^5%^, *T*_d_^10%^, and *R*_w_ values ranging from 523 °C to 533 °C, 540 °C to 551 °C, and 54.1% to 53.2%, respectively. Additionally, when copolymerized at a 4:6 ratio, the *T*_d_^5%^, *T*_d_^10%^, and *R*_w_ values ranged from 512 °C to 518 °C, 523 °C to 530 °C, and 56.3% to 55.2%, respectively. These findings suggest that the other three isomeric PIs also maintain high thermal stability, with no significant differences in thermal stability observed among the four isomeric PIs.

### 3.4. Mechanical Properties

The mechanical properties of the PI films are crucial performance indicators, and all films were tested using an electronic universal testing machine. The mechanical properties are summarized in Table 3. As the copolymerization molar ratio of 5a increased from 0 to 1:9, 2:8 and 4:6, the maximum tensile strength initially increased and then decreased, while the initial modulus consistently increased, and the elongation at break decreased. This behavior is attributed to the enhanced rigidity and twist of the polymer chains introduced by the spirobis(indene)-bis(benzoxazole) frameworks, which increased the modulus but reduced ductility. The tensile strength increase at first reflects the beneficial effect of increased chain rigidity, while the subsequent decline indicates a loss of toughness leading to brittle failure due to excessive rigidity. For the other three isomeric diamines (5aa, 5b, 5bb) copolymerized at a 1:9 molar ratio, the corresponding tensile strengths were 133.8 MPa, 131.6 MPa and 125.3 MPa, with initial moduli of 2.5 GPa, 2.6 GPa and 3.5 GPa, and elongation at break values of 16.5%, 15.8% and 14.7%, respectively. Similarly, at a 4:6 molar ratio, the tensile strengths were 105.2 MPa, 101.3 MPa and 98.3 MPa, with elastic moduli of 4.1 GPa, 3.4 GPa and 4.7 GPa, and elongation at break values of 3.3%, 4.4% and 2.8%. All other isomeric films exhibited similar mechanical properties. These results indicate that the PI films derived from the copolymerization of spirobis(indene)-bis(benzoxazole) diamines with ODA and PMDA exhibit not only excellent thermal properties, but also good mechanical performance.

### 3.5. Dielectric Properties

PIs are widely utilized in the fields of integrated circuits and insulation materials due to their excellent insulating properties. The *D*_k_ values of all PI films at high frequencies were measured using a PNAN5227B vector network analyzer (Keysight, Santa Rosa, CA, USA) under controlled conditions (25 ± 1 °C, 40% RH), with the results summarized in Table 4. The Kapton film (ODA-PMDA) was initially measured, showing a dielectric constant of approximately 3.51 at 10 GHz. When 5a and ODA were copolymerized with PMDA at molar ratios of 1:9, 2:8 and 4:6, the resulting PI films exhibited a significant reduction in *D*_k_ values. The rigid, bulky semi-aliphatic spirobis(indene) structure effectively decreased the molar polarization of the PIs, reducing the *D*_k_ values to approximately 3.37, 3.16 and 3.08 at 10 GHz, respectively. For the other three isomeric diamines (5aa, 5b and 5bb), when copolymerized with ODA at molar ratios of 1:9 and 4:6 with PMDA, the resulting PI films showed no notable differences in *D*_k_ values compared to the corresponding 5a series PI films. As the frequency increased (40 GHz, 60 GHz), the *D*_k_ values of all PI films exhibited a slight decrease. This trend is primarily attributed to the interplay of dipolar and ionic polarization effects. At higher frequencies, as the frequency increases, the effect of reduced dipolar polarization outweighs that of the emerging ionic polarization, leading to a minor decrease in *D*_k_ values across all polymer films [50,51,52,53]. In conclusion, incorporating spirobis(indene)-bis(benzoxazole) structures into the PI backbone represents an effective strategy for reducing the dielectric constant of PI films.

Furthermore, the *D*_k_ of PI films is closely related to their water absorption rate. Therefore, the water absorption behavior of all films was further measured, and the data are presented in Table 4. With the introduction of the 5a monomer, the water absorption rates (*A*) of films decreased from 2.62 (Kapton) to 2.44 (1:9), 2.28 (2:8) and 1.93 (4:6), respectively. A similar decreasing trend was observed for the other three isomeric polymers. This is primarily attributed to the hydrophobic nature of the spirobis(indene) group, which is semi-aliphatic. Additionally, the incorporation of the spirobis(indene)-bis(benzoxazole) structures reduced the percentage of hydrophilic amide ring in the PI structure, leading to a decrease in water absorption, which is consistent with the observed trend in the *D*_k_.

## 4. Conclusions

This study successfully synthesized four spirobis(indene)-bis(benzoxazole) diamines (5a, 5aa, 5b and 5bb), which were copolymerized with ODA and PMDA in varying molar ratios to create a series of PI films. The introduction of the distorted rigid spirobis(indene) units and the linear rigid benzoxazole framework effectively enhances the rigidity of the PI molecular chains while restricting their mobility, resulting in significantly improved thermal stability and mechanical properties of the Kapton-type films. Compared to Kapton, when the molar ratio of 5a to ODA is only 1:9, the *T*_g_, modulus, and dielectric constant of 5a1-ODA9-PMDA can be increased from 396 °C, 1.6 GPa and 3.50 to 467 °C, 2.5 GPa and 3.31, respectively. At a molar ratio of 4:6, these values further reach 520 °C, 4.7 GPa and 2.87. Moreover, the incorporation of the spirobis(indene)-bis(benzoxazole) structures maintains excellent high-temperature dimensional stability and mechanical performance. This research provides a feasible strategy for the preparation of ultra-heat-resistant, high-modulus, and low-dielectric polymer films, offering significant potential applications in high-temperature fields such as aerospace and flexible substrates.

## Figures and Tables

**Table 1:** Molecular weights and physical parameters of the Kapton-type PIs.

PAAs	*M*_n_ (×10^4^) *^a^*	*M*_w_ (×10^4^) *^a^*	PDI *^b^*	*ρ* (g m^−3^)	Thickness (μm)
ODA-PMDA (Kapton)	6.4	11.4	1.8	1.402	34
5a1-ODA9-PMDA	8.9	17.5	2.0	1.391	32
5a2-ODA8-PMDA	9.4	19.1	2.0	1.375	36
5a4-ODA6-PMDA	9.7	20.2	2.1	1.358	32
5a5-ODA5-PMDA	8.8	20.5	2.3	-	Brittle
5aa1-ODA9-PMDA	7.7	15.3	1.9	1.395	31
5b1-ODA9-PMDA	7.3	14.6	2.0	1.393	45
5bb1-ODA9-PMDA	5.9	11.2	1.9	1.389	31
5aa4-ODA6-PMDA	5.3	12.3	2.3	1.359	36
5b4-ODA6-PMDA	7.2	15.8	2.2	1.361	31
5bb4-ODA6-PMDA	6.1	12.7	2.1	1.357	39

*^a^* Molecular weight of the polymers, unit g mol^−1^. *^b^* PDI: Polymer dispersity index.

**Table 2:** Thermal properties of Kapton-type PI films.

Polyimide	*T*_g_ (°C) *^a^*	*T*_d_^5%^ (°C) *^b^*	*T*_d_^10%^ (°C) *^c^*	*R*_w_ (%) *^d^*	CTE (ppm/K) *^e^*
ODA-PMDA	396	558	581	56.8	32
5a1-ODA9-PMDA	467	530	549	56.7	34
5a2-ODA8-PMDA	500	525	539	54.3	36
5a4-ODA6-PMDA	>520	522	535	56.2	38
5aa1-ODA9-PMDA	454	532	551	54.1	34
5b1-ODA9-PMDA	463	533	554	54.7	36
5bb1-ODA9-PMDA	444	523	540	53.2	39
5aa4-ODA6-PMDA	>510	515	526	55.2	43
5b4-ODA6-PMDA	509	512	523	56.1	45
5bb4-ODA6-PMDA	504	518	530	56.3	42

*^a^ T*_g_: glass transition temperature tested by DMA. *^b^ T*_d_^5%^: temperatures at 5% weight loss. *^c^ T*_d_^10%^: temperatures at 10% weight loss. *^d^* Residual weight retention when heated to 800 °C in N_2_. *^e^* CTE: measured with TMA in the temperature range of 50–300 °C.

**Table 3:** Mechanical properties of Kapton-type PI films.

Polyimide	*σ* (MPa) *^a^*	*E* (GPa) *^b^*	*ε* (%) *^c^*
ODA-PMDA	127.3 ± 5.3	1.6 ± 0.3	33.1 ± 3.8
5a1-ODA9-PMDA	132.6 ± 6.3	2.5 ± 0.4	16.6 ± 1.2
5a2-ODA8-PMDA	118.3 ± 9.3	3.3 ± 0.3	5.8 ± 1.5
5a4-ODA6-PMDA	97.5 ± 7.4	4.7 ± 0.5	2.9 ± 0.9
5aa1-ODA9-PMDA	133.8 ± 6.2	2.4 ± 0.4	16.5 ± 1.9
5b1-ODA9-PMDA	131.6 ± 10.2	2.6 ± 0.5	15.8 ± 2.1
5bb1-ODA9-PMDA	125.3 ± 7.9	3.5 ± 0.6	14.7 ± 1.7
5aa4-ODA6-PMDA	105.2 ± 6.9	4.1 ± 0.3	3.3 ± 0.5
5b4-ODA6-PMDA	101.3 ± 7.8	3.4 ± 0.4	4.4 ± 0.6
5bb4-ODA6-PMDA	98.3 ± 8.4	4.7 ± 0.4	2.8 ± 0.5

*^a^* Tensile strength; *^b^* initial modulus; *^c^* elongation at break.

**Table 4:** Electronic properties of the Kapton-type PI films.

Polyimide	*D*_k_ *^a^*			*A* (%) *^b^*
	10 GHz	40 GHz	60 GHz	
ODA-PMDA	3.51	3.48	3.50	2.62
5a1-ODA9-PMDA	3.37	3.35	3.31	2.44
5a2-ODA8-PMDA	3.16	3.15	3.13	2.28
5a4-ODA6-PMDA	3.08	2.89	2.87	1.93
5aa1-ODA9-PMDA	3.34	3.35	3.34	2.48
5b1-ODA9-PMDA	3.38	3.38	3.37	2.45
5bb1-ODA9-PMDA	3.34	3.33	3.33	2.47
5aa4-ODA6-PMDA	3.06	2.95	2.92	1.95
5b4-ODA6-PMDA	3.02	2.88	2.87	1.98
5bb4-ODA6-PMDA	2.98	2.89	2.96	1.96

*^a^ D*_k_: dielectric constant. *^b^* A: absorption rate.

## Data Availability

The original contributions presented in the study are included in the article and supplementary material, further inquiries can be directed to the corresponding author.

## Supplementary Materials

The following supporting information can be downloaded at: https://www.mdpi.com/article/10.3390/polym17070832/s1, Scheme S1: Synthetic route of four spirobis(indene)-bis(benzoxazole) containing diamine; Figure S1: Picture of PI films; Figure S2: DMA curves of Kapton-type PIs. The Supporting Information is available free of charge. References [54,55,56,57,58,59,60,61,62] are cited in the supplementary materials.
