# Supplementary material for: Exceptionally High-Temperature-Resistant Kapton-Type Polyimides with Tg > 520 °C: Synthesis via Incorporation of Spirobis(indene)-bis(benzoxazole)-Containing Diamines
Source: Polymers (Basel). 2025 Mar 21;17(7):832. doi: 10.3390/polym17070832 (PMC11991488; doi:10.3390/polym17070832)
Supplement: Supplementary file 1 [file polymers-17-00832-s001.zip › polymers-3537422-supplementary.pdf]

polymers

MDPI

Supporting Information

Exceptionally High-Temperature-Resistant Kapton-Type Polyimides with T_g > 520 °C: Synthesis via Incorporation of Spirobis(Indene)-Bis(Benzoxazole)-Containing Diamines

Peng Xiao [1,2], Xiaojie He [2] and Qinghua Lu [3,*]

[1] Institute of Micro/Nano Materials and Devices, Ningbo University of Technology, Ningbo 315211, China; pengxiao2020@tongji.edu.cn

[2] School of Chemical Science and Technology, Tongji University, Siping Road No. 1239, Shanghai 200092, China; 2011327@tongji.edu.cn

[3] State Key Laboratory of Synergistic Chem-Bio Synthesis, School of Chemistry and Chemical Engineering, Shanghai Jiao Tong University, Dongchuan

1. Materials

Nitric acid (98%) and hydrazine hydrate ($N_2H_4{\cdot}H_2O$, 80%) were provided by Sinopharm Chemical Reagent Co., Ltd. (Shanghai, China). Bisphenol A (98%) and methanesulfonic acid (96%) were obtained from Tokyo Chemical Industry Co., Ltd. (Tokyo, Japan). 4-Aminobenzoic acid (99.0%), 3-aminobenzoic acid (99.0%), acetic acid (99.5%), polyphosphoric acid (85%), phosphorus pentoxide (99.5%), palladium on carbon (Pd/C, 10 wt%), sodium bicarbonate (99.5%), triethylamine (99%), deuterated chloroform (99.8%), deuterated dimethyl sulfoxide (99.0%), coarse silica gel (100–200 mesh), fine silica gel (300–400 mesh), absolute ethanol (analytical grade, 99.8%), ethyl acetate (analytical grade, 99.7%), dichloromethane (analytical grade, 99.5%), and petroleum ether (analytical grade, 99.8%) were purchased from Adamas Reagent Co., Ltd. (Shanghai, China). N,N-Dimethylacetamide (water content <50 ppm) was acquired from J&K Scientific Ltd. (Hong Kong). Pyromellitic dianhydride (PMDA, 99.5%) and 4,4′-oxydianiline (ODA, 99.5%) were supplied by ChinaTech (Tianjin) Chemical Co., Ltd., Tianjin, China. All reagents and solvents were used as received without further purification.

2. Characterization

^1H and ^{13}C NMR spectra of the compounds were obtained on a Bruker AVANCE III HD 600 spectrometer (Bruker BioSpin, Ettlingen, Germany) at 600 MHz and 150 MHz, respectively, from their solutions in DMSO-d_6 or $CDCl_3$. Chemical shifts are quoted relative to tetramethylsilane (TMS). Gel permeation chromatography (GPC) was performed on a HLC-8320GPC (TOSOH, Tokyo, Japan) equipped with a TSKgel SuperMultiporeHZ column and a laser-refractive index detector using DMAc containing 0.03 mol/L lithium bromide (LiBr) and 0.03 mol/L H_3PO_4 as eluent. The number-average (M_n) and weight-average (M_w) molecular weights were estimated by using a polystyrene standard calibration curve. Infrared spectra of PI films were acquired in attenuated total reflectance (ATR) mode on a Nicolet 6700 FTIR spectrometer (PerkinElmer, Inc., Waltham, MA, USA) over the range 4000 cm^{-1}-500 cm^{-1} by accumulating 16 scans. Dynamic mechanical analysis (DMA) was recorded on a TA Instruments Q800 (New Castle, DE, USA) under an nitrogen atmosphere at 5 °C/min with a frequency of 1 Hz. Thermogravimetric analysis (TGA) was

 https://doi.org/10.3390/polym17070832

measured by a Perkin Elmer Pyris analyzer (USA) under nitrogen at 10 °C/min. The coefficient of thermal expansion (CTE) was collected by a TA Instruments Q400 analyzer (USA) with a static load of 0.05 N at 5 °C/min from 50-300°C under nitrogen, the values were calculated by the second heating curves without residual stress. Film samples (100 mm × 10 mm) were strained at 5 mm/min on a CMT1104 universal electromechanical tester (SUST., Xianyang, China) to record their mechanical properties. The density of the polymers were calculated with the formula, $\rho = M/V$, where M was the weight of the and V was obtained from the accurate measurement of the thickness and area. Wide-angle X-ray scattering of the film samples were conducted on a Bruker D8 DISCOVER apparatus at a scanning rate of 5 °/min from 5 to 45°, the film were loaded on a zero-background silicon substrate.

In water absorption tests, PI films held at 80°C for 2 h were immersed into deionized water at 25°C for 48 h. Then, water on the film surface after taking out was cleaned with absorbent paper. The weights of the films were measured immediately. The water absorption ratio (P) was calculated from Equation (S1):

$$A(\%) = \frac{m_1 - m_0}{m_1} \times 100\% \tag{S1}$$

where m_0 and m_1 are the film mass before and after soaking, respectively.

3. Computational simulation of the T_g

The simulation and computational analyses of the relevant polymers in this study were conducted using the Material Studio 2019 software. Based on our team's previous research reports,[1,2] the process primarily comprises model construction, structural optimization and equilibration, and characterization methods for the molecularly simulated structures.

Model Construction: First, a PI molecular chain was constructed, with a degree of polymerization set to 15.[3] The molecular chain was then structurally optimized, and a polymer system was constructed using the Amorphous Cell (AC) module,[4] where the PI system model consisted of 12 chains. Given the inherent randomness of the Monte Carlo method used for modeling, five unit cells were established for each PI structure. The structure with the lowest energy among the five was selected for subsequent equilibration.

Structural Optimization and Equilibration Process: This stage mainly includes the annealing process and dynamic equilibration. First, molecular dynamics simulations were conducted under an NPT ensemble (constant pressure and temperature) for 50 ps to obtain a structure with reasonable density. Next, an NVT ensemble (constant volume and temperature) was employed for 300 ps to perform annealing dynamics simulations, seeking the global energy minimum configuration. Following the annealing dynamics, the dynamic equilibration process was conducted by performing 400 ps of NVT molecular dynamics simulations at 298 K to eliminate internal stress, with temperature control achieved using the Nose method.[5] Finally, based on the initial velocity from the previous step, molecular dynamics simulations were carried out under a pressure of 0.0001 GPa and at 298K using the Berendsen pressure control method for 800 ps, achieving a stable equilibrium density.[6] Throughout the molecular dynamics (MD) simulations, the COMPASS II force field was used.[7] When the temperature fluctuation range was within 3%, and the density fluctuation in the final frames did not exceed 0.02 g/cm^3, the model was considered equilibrated.[8]

The simulation of the polymer's T_g is based on the free volume theory proposed by Fox and Flory,[9-11] which is used to explain the glass transition behavior of polymers. Below the glass transition temperature, the polymer exists in a glassy state, and as the temperature increases, the changes in the polymer's free volume or density are minimal. However,

when the temperature reaches the glass transition temperature, the free volume or density of the polymer undergoes a sudden change. Therefore, by plotting the density/free volume versus temperature curve and fitting the curve, the point of discontinuity in the polymer's density with temperature can be determined, which corresponds to the theoretically simulated T_g value. The process is as follows: Using Material Studio 2019 software, five PI boxes, each containing 12 molecular chains (degree of polymerization = 15), were constructed using the Amorphous Cell module. The polymer's density after equilibration was close to experimental values, ranging between 1.2-1.4 g/cm^3, indicating that the model is reasonable. The geometric optimization of these models was performed using the Smart algorithm in the Forcite module. A 50 ps NPT dynamics simulation was first conducted to obtain a structure with reasonable density. This was followed by a 400 ps molecular dynamics simulation under the NVT ensemble at 298 K to eliminate internal stresses. Finally, an 800 ps molecular dynamics simulation was performed under the NPT ensemble at 0.0001 GPa and 298 K, using the initial velocities from the previous step, to obtain a stable equilibrium density. Based on this, a temperature range from 500 K to 1000 K was set with a 20 K interval to obtain the corresponding densities at each temperature. The next step was to plot the temperature-density curve of the polymer. Throughout the MD simulations, the COMPASS II force field was used.

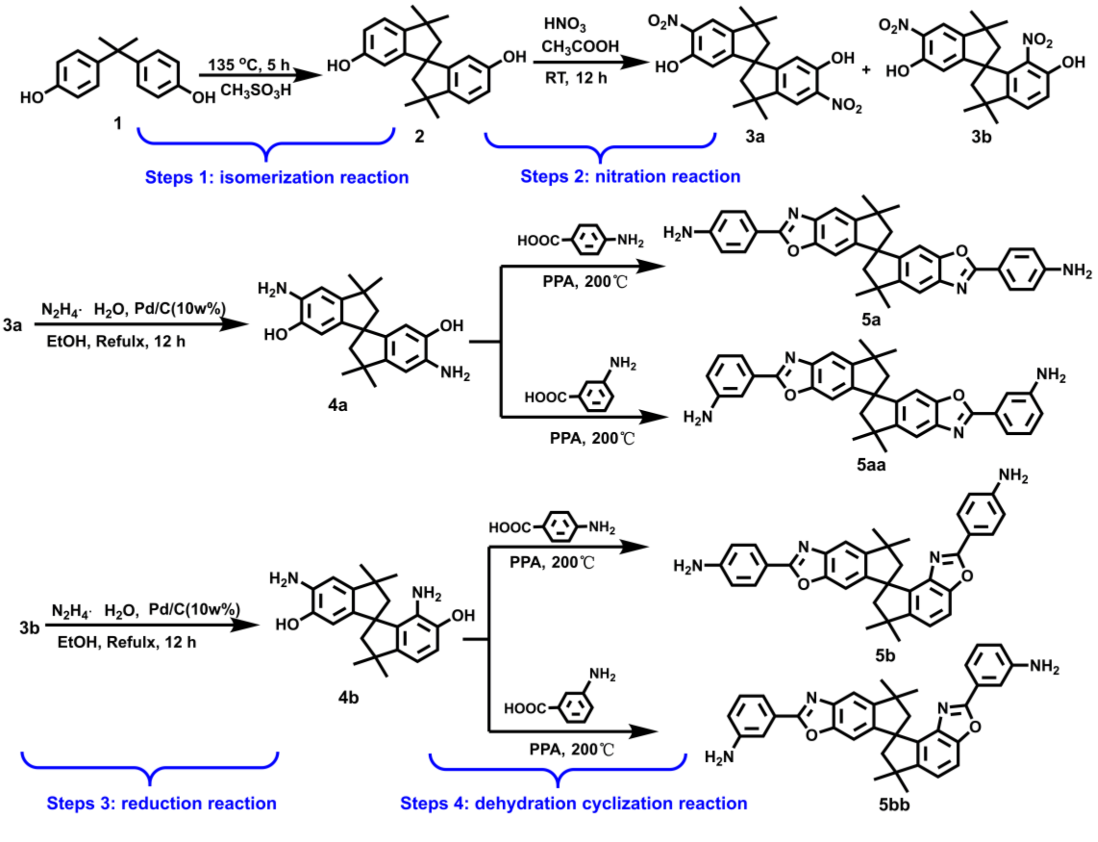

Scheme S1. Synthetic route of four spirobis(indene)-bis(benzoxazole) containing diamine.

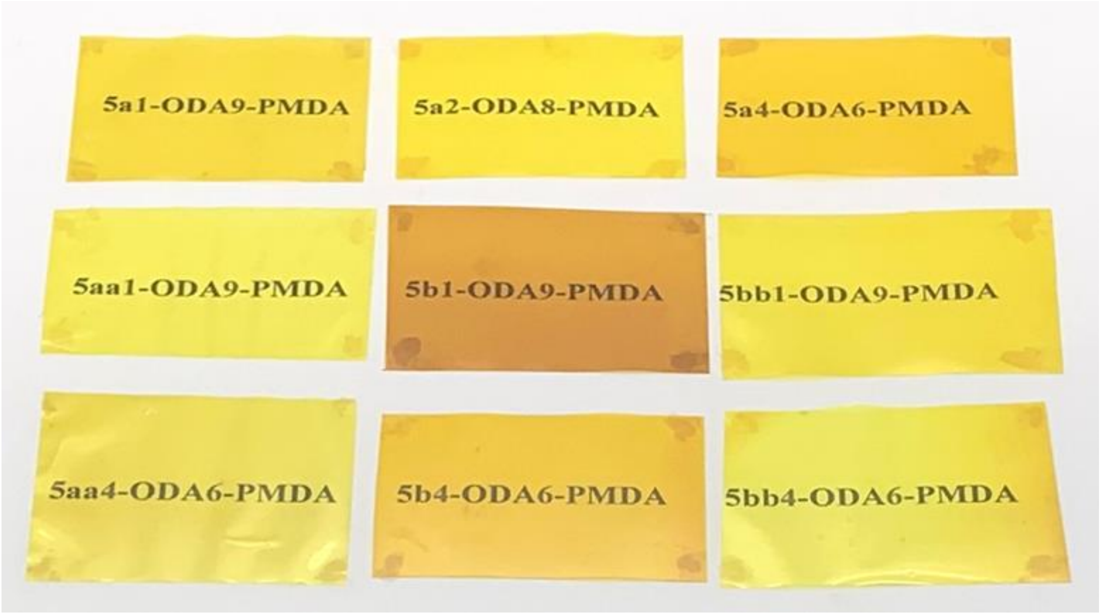

Figure S1. Picture of PI films.

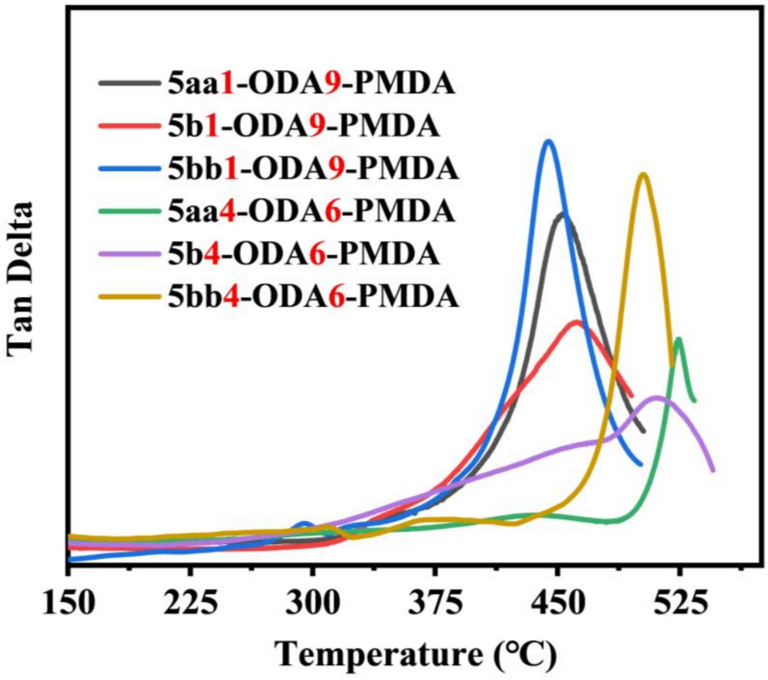

Figure S2. DMA curves of Kapton-type PIs.

References

1. Ma, X.; Zheng, F.; van Sittert, C.; Lu, Q. Role of Intrinsic Factors of Polyimides in Glass Transition Temperature: An Atomistic Investigation. *J. Phys. Chem. B* **2019**, *123*, 8569.
2. Xiao, P.; He, X.; Zheng, F.; Lu, Q. Super-heat resistant, transparent and low dielectric polyimides based on spirocyclic bisbenzoxazole diamines with Tg > 450 °C. *Polym. Chem.* **2022**, *13*, 3660.
3. Hofman, D.; Ulbrich, J.; Fritsch, D.; Paul, D. Molecular modelling simulation of gas transport in amorphous polyimide and poly(amide imide) membrane materials. *Polymer* **1996**, *37*, 4773.
4. Abe, A.; Jernigan, R.L.; Flory, P.J. Conformational Energies of n-Alkanes and the Random Configuration of Higher Homologs Including Polymethylene. *J. Am. Chem. Soc.* **1966**, *88*, 631.
5. Evans, D.J.; Holian, B.L. The Nose–Hoover thermostat. *J. Chem. Phys.* **1985**, *83*, 4069.
6. Faller, R.; de Pablo, J.J. Constant pressure hybrid Molecular Dynamics–Monte Carlo simulations. *J. Chem. Phys.* **2002**, *116*, 55.

7. Liu, H.Y.; Fang, C.H.; Fang, Y.; Zhou, Y.Q.; Ge, H.W.; Zhu, F.Y.; Sun, P.C.; Miao, J.T. Characterizing Ni(II) hydration in aqueous solution using DFT and EXAFS. *J. Mol. Model.* **2015**, *22*, 2.
8. Shi, W.-Y.; Ding, C.; Yan, J.-L.; Han, X.-Y.; Lv, Z.-M.; Lei, W.; Xia, M.-Z.; Wang, F.-Y. Molecular dynamics simulation for interaction of PESA and acrylic copolymers with calcite crystal surfaces. *Desalination* **2012**, *291*, 8.
9. Fox, T.G., Jr.; Flory, P.J. Second-Order Transition Temperatures and Related Properties of Polystyrene. I. Influence of Molecular Weight. *J. Appl. Phys.* **1950**, *21*, 581.
10. Fox, T.G., Jr.; Flory, P.J. Further Studies on the Melt Viscosity of Polyisobutylene. *J. Phys. Chem.* **1951**, *55*, 221.
11. Fox, T.G.; Flory, P.J. The glass temperature and related properties of polystyrene. Influence of molecular weight. *J. Polym. Sci.* **1954**, *14*, 315.

Disclaimer/Publisher's Note: The statements, opinions and data contained in all publications are solely those of the individual author(s) and contributor(s) and not of MDPI and/or the editor(s). MDPI and/or the editor(s) disclaim responsibility for any injury to people or property resulting from any ideas, methods, instructions or products referred to in the content.
